# Supplementary figures and images for: In utero adeno-associated virus (AAV)-mediated gene delivery targeting sensory and supporting cells in the embryonic mouse inner ear
Source: PLoS One. 2024 Jul 19;19(7):e0305742. doi: 10.1371/journal.pone.0305742 (PMC11259301; doi:10.1371/journal.pone.0305742)

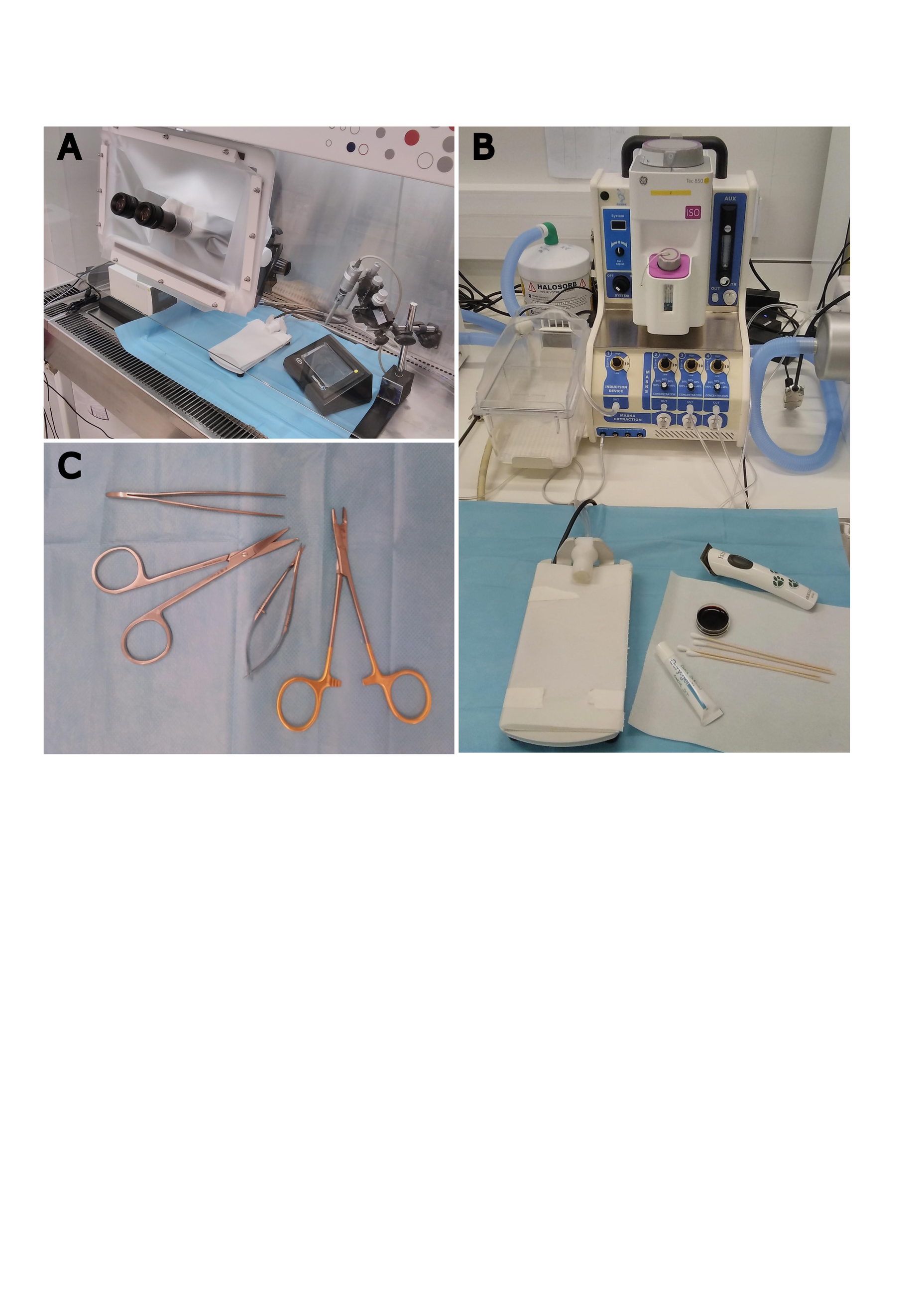

Supplement: S1 Fig — (A) Safety cabinet equipped with a binocular in the operatory area. (B) The preparatory area equipped with an anesthesia set up. In this area the pregnant dam is shaved in the stomach area after which an antiseptic solution of vetedine is applied. A solution of ocrygel is applied to both eyes. (C) Graefe forceps, ball-tipped scissors, microfine scissors as well as a needle holder used for the ventral laparotomy, as well as suturing. (TIF) [file pone.0305742.s001.tif]

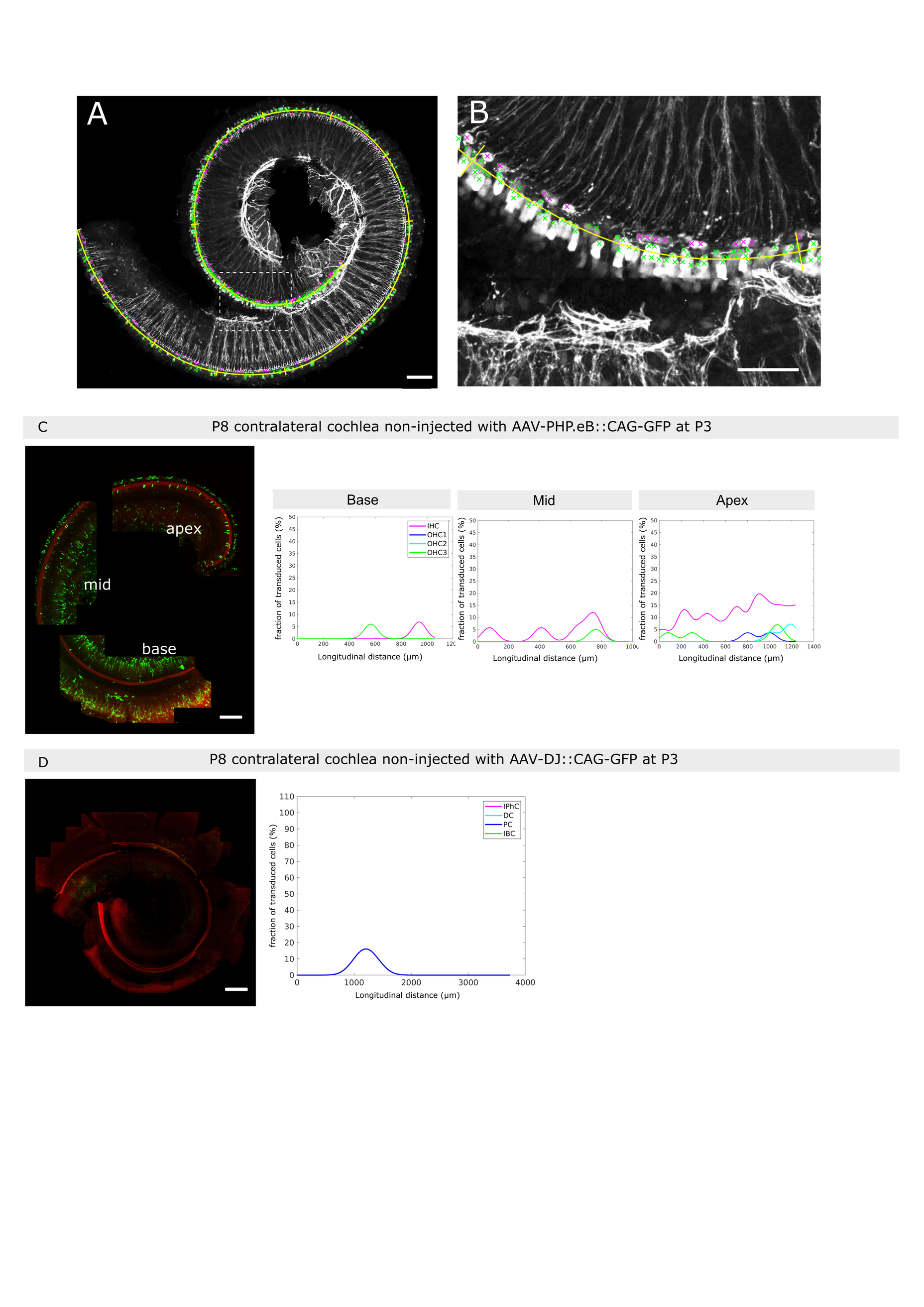

Supplement: S2 Fig — (A) Confocal maximum projection image of a P0 cochlea injected with AAV-PHP.eB::CAG-GFP on E13.5. Sensory cells expressing GFP are in white. The longitudinal cochlear axis selected is shown (yellow spiral curve), together with the selected transduced cells with crosses in magenta for IHCs and in green OHCs. (B) Detail on the area delineated by the tireted rectangle in (A). (C,D) P8 contraleral cochleas from P3 injected pups. Confocal maximum intensity projection images of right cochleas extracted from pups injected unilaterally in the left ear with AAV-PHP.eB::CAG-GFP in (C) and AAV-DJ::CAG-GFP in (D). The graphs on the right of the images show the longitudinal transduction rate profiles estimated for each of the imaged cochlear fragments. Scale bars 100 μm. (TIF) [file pone.0305742.s002.tif]

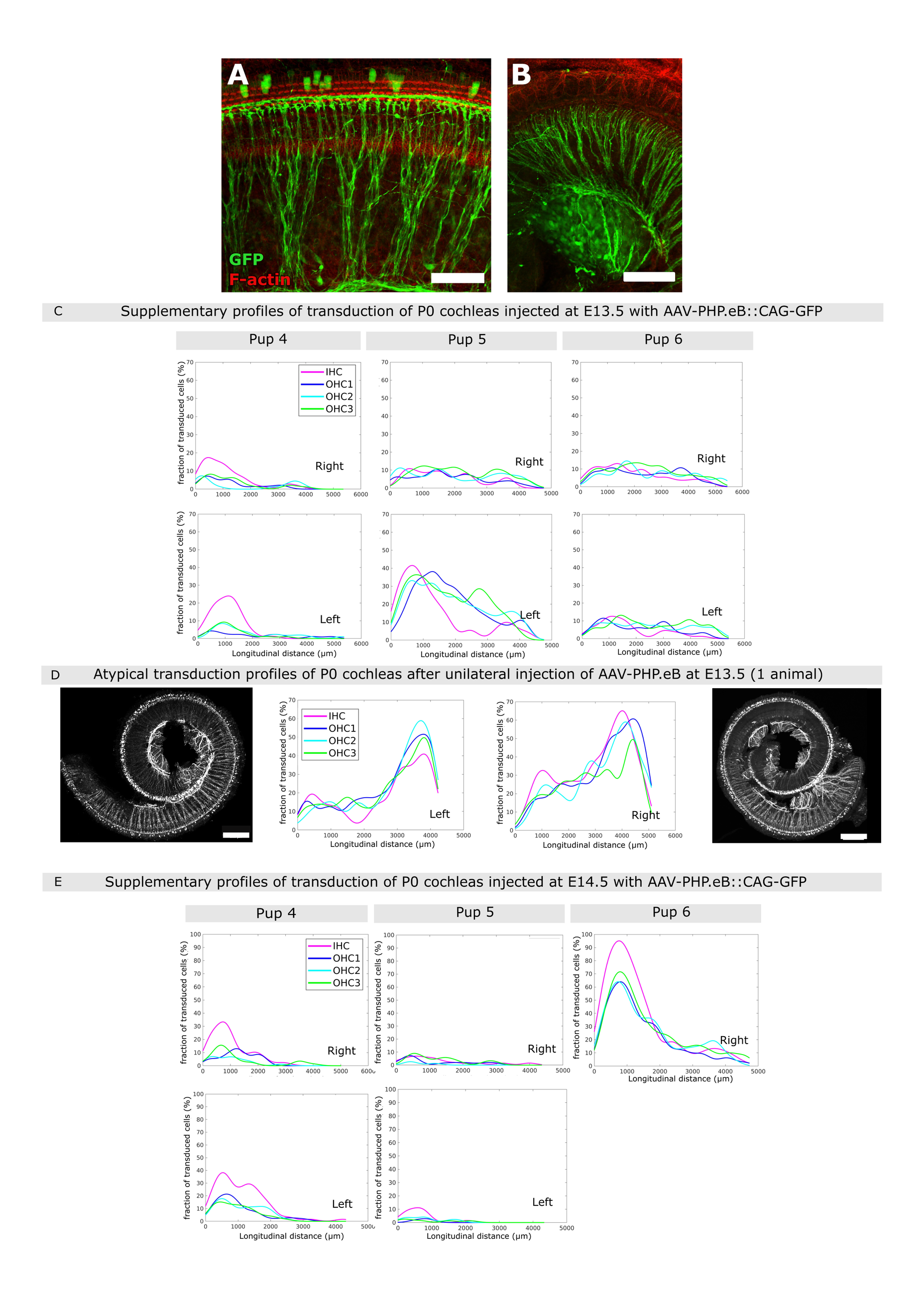

Supplement: S3 Fig — (A,B) Auditory nerve fibers (A) and ganglion-like cells (B) expressing GFP, in P0 cochlea injected at E13.5. Scale bar 100 μm. (C) Additional representative graphs of the longitudinal transduction profiles measured in the right and left cochleas of 3 individual pups injected at E13.5, and 3 pups injected at E14.5. (D) Longitudinal transduction profiles measured in the right and left cochleas of an atypical individual showing a reversed transduction profile. (E) Longitudinal transduction rate profiles observed in P0 cochleas from three additional pups after unilateral injection of AAV-PHP.eB at E14.5. (TIF) [file pone.0305742.s003.tif]

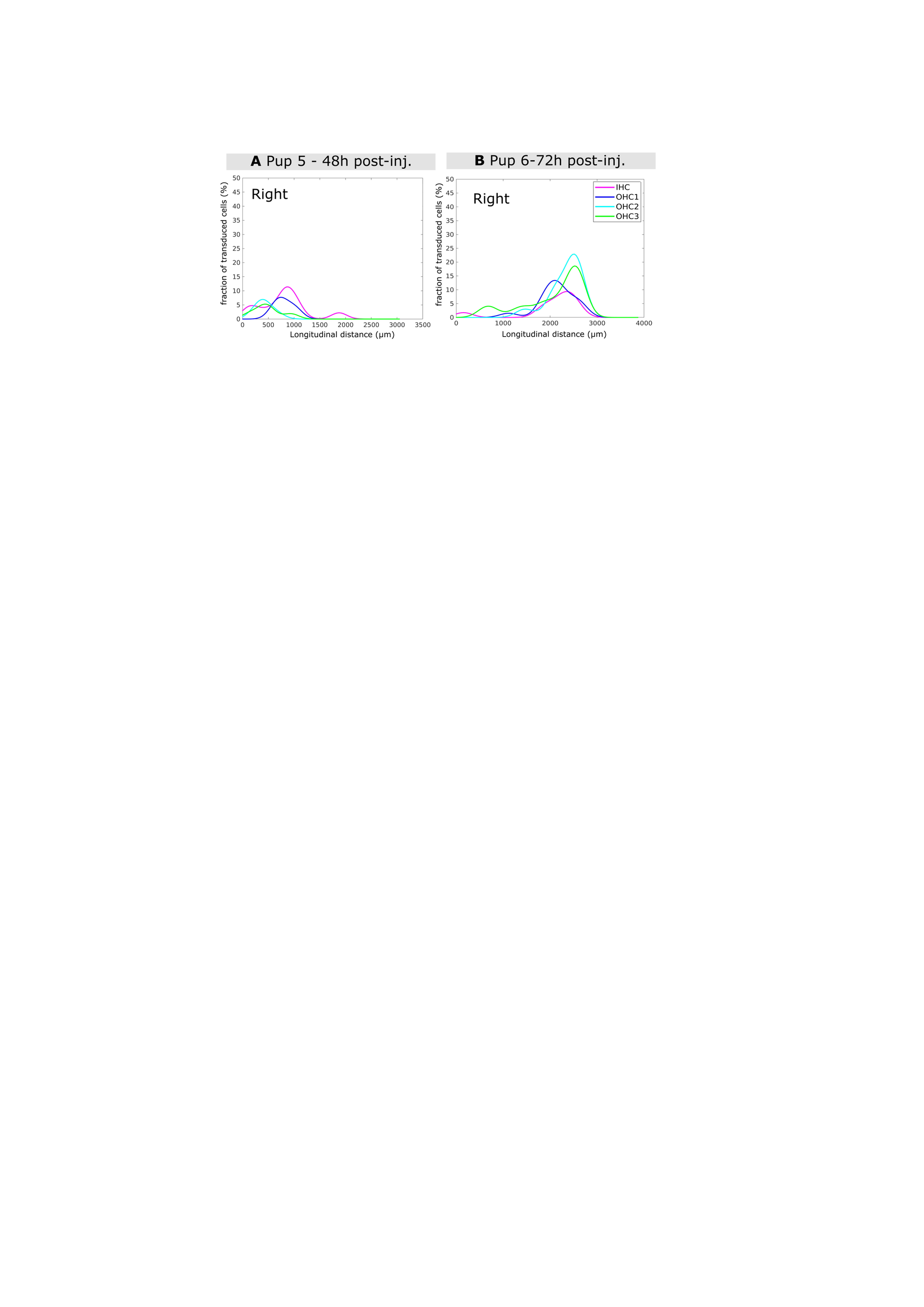

Supplement: S4 Fig — Longitudinal transduction rate profiles observed in the right cochleas from two additional pups dissected 48 hours (at E15.5) (A) and 72 hours (at E16.5) (B) post-injection, after unilateral injection of AAV-PHP.eB at E13.5. (TIF) [file pone.0305742.s004.tif]

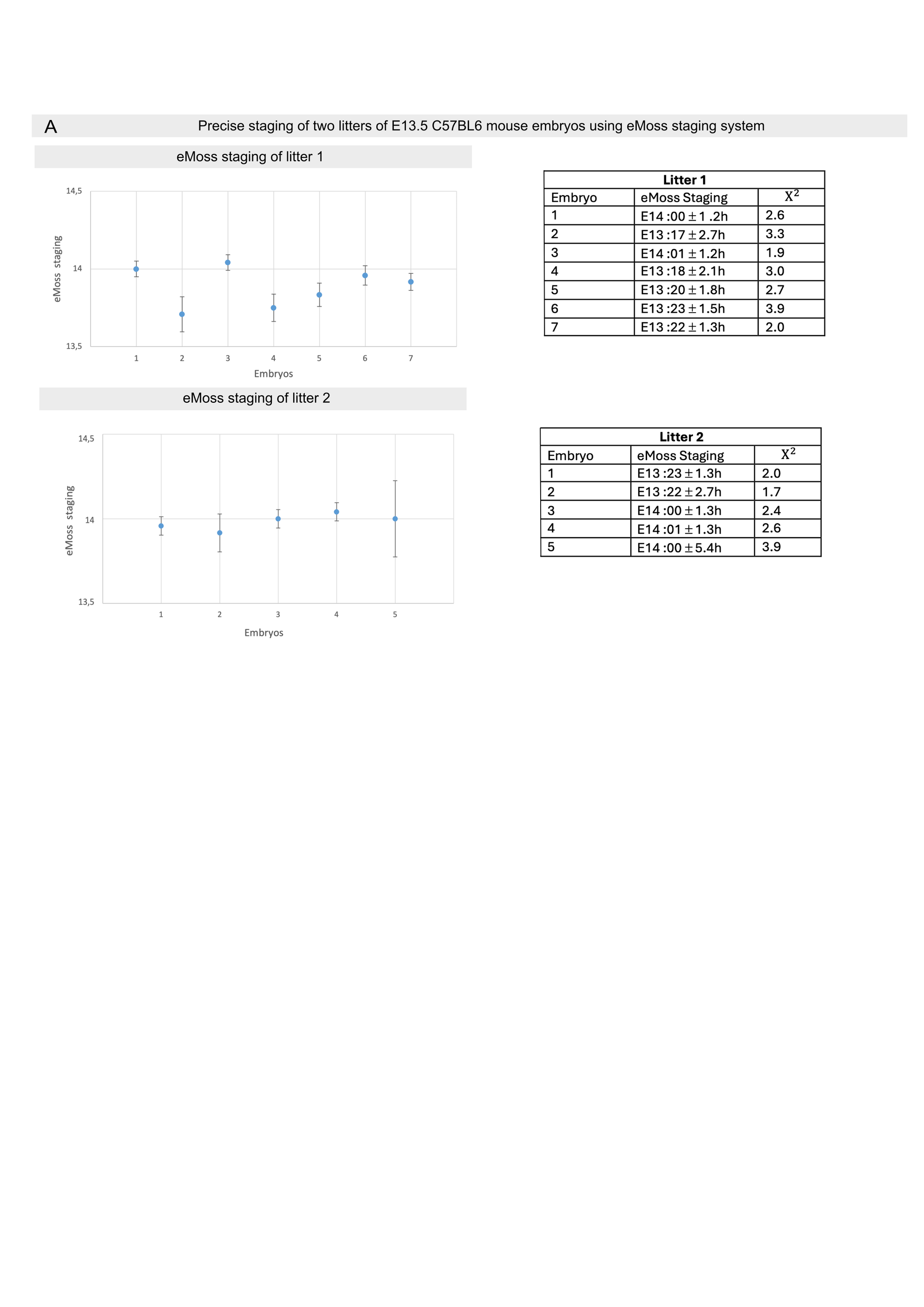

Supplement: S5 Fig — On the left are shown plots of eMoss staging results based on hindlimbs measurements on two litters (1 and 2) of mouse embryos initially staged E13.5 based on the day when the vaginal plug was observed. The tables on the right contain the corresponding staging values estimated by eMoss (given in hours following the actual embryonic day matching the hindlimbs measurements). Only the results for which the χv2 values were bellow 5 were considered as reliable and were used for the analysis [20]. (TIF) [file pone.0305742.s005.tif]
